# State-Targeting Stabilization of Adenosine A_2A_ Receptor by Fusing a Custom-Made De Novo Designed α-Helical Protein

**DOI:** 10.3390/ijms222312906

**Published:** 2021-11-29

**Authors:** Masaya Mitsumoto, Kanna Sugaya, Kazuki Kazama, Ryosuke Nakano, Takahiro Kosugi, Takeshi Murata, Nobuyasu Koga

**Affiliations:** 1 Department of Structural Molecular Science, School of Physical Sciences, SOKENDAI, The Graduate University for Advanced Studies, Shonan Village, Hayama 240-0193, Kanagawa, Japan; sanbon@ims.ac.jp (M.M.); takahirokosugi@ims.ac.jp (T.K.); 2 Research Center of Integrative Molecular Systems, Institute for Molecular Science (IMS), National Institutes of Natural Sciences (NINS), Okazaki 444-8585, Aichi, Japan; 3 Department of Chemistry, Graduate School of Science, Chiba University, Chiba 263-8522, Japan; k.sugaya@chiba-u.jp (K.S.); breeze.shine1010@icloud.com (K.K.); ryo2009@friend.ocn.ne.jp (R.N.); 4 Protein Design Group, Exploratory Research Center on Life and Living Systems (ExCELLS), National Institutes of Natural Sciences (NINS), Okazaki 444-8585, Aichi, Japan; 5 Membrane Protein Research Center, Chiba University, Chiba 263-8522, Japan

**Keywords:** computational protein design, protein stabilization, de novo designed protein, fusion partner protein, G-protein coupled receptor, adenosine A_2A_ receptor

## Abstract

G-protein coupled receptors (GPCRs) are known for their low stability and large conformational changes upon transitions between multiple states. A widely used method for stabilizing these receptors is to make chimeric receptors by fusing soluble proteins (i.e., fusion partner proteins) into the intracellular loop 3 (ICL3) connecting the transmembrane helices 5 and 6 (TM5 and TM6). However, this fusion approach requires experimental trial and error to identify appropriate soluble proteins, residue positions, and linker lengths for making the fusion. Moreover, this approach has not provided state-targeting stabilization of GPCRs. Here, to rationally stabilize a class A GPCR, adenosine A_2A_ receptor (A_2A_R) in a target state, we carried out the custom-made de novo design of α-helical fusion partner proteins, which can fix the conformation of TM5 and TM6 to that in an inactive state of A_2A_R through straight helical connections without any kinks or intervening loops. The chimeric A_2A_R fused with one of the designs (FiX1) exhibited increased thermal stability. Moreover, compared with the wild type, the binding affinity of the chimera against the agonist NECA was significantly decreased, whereas that against the inverse agonist ZM241385 was similar, indicating that the inactive state was selectively stabilized. Our strategy contributes to the rational state-targeting stabilization of GPCRs.

## 1. Introduction

G-protein coupled receptors (GPCRs) are the largest membrane protein family in the human genome [1]. GPCRs mediate signals related to diverse physiological functions; therefore, these receptors are major drug targets [2]. GPCRs are in equilibrium between multiple states [3] and are known for their innate instability, which causes difficulties in sample preparation, functional assays, or structure determinations. Therefore, methods for stabilization have been developed, including alanine scanning [4,5], directed evolution [6], and rational mutation [7,8].

The fusion partner strategy has been widely used, in which the intracellular loop 3 (ICL3) connecting the transmembrane helices 5 and 6 (TM5 and TM6) is replaced with soluble protein domains, such as T4-lysozyme [9,10], apocytochrome b562RIL (BRIL) [11,12], rubredoxin [13], and glycogen synthetase [14]. However, these fusion partner proteins and residue positions for fusion have been identified through experimental trial and error. Moreover, stabilizing GPCRs in a specific state using the fusion approach, which is useful for screening out state-dependent ligands and antibodies [15,16], has not been achieved.

Recently, principles for designing protein structures from scratch have been developed, which made it possible to create a wide range of new protein structures with high thermal stability [17,18,19]. We previously developed a method to create a diverse set of all-α protein structures ranging from bundle-like topologies with parallel-aligned helices to complicated ones with irregularly arranged helices [20]. Using the developed method, we sought to rationally design fusion partner proteins customized for not only thermally stabilizing GPCRs but also stabilizing them in a target state compared to the other states.

**Figure 1:**
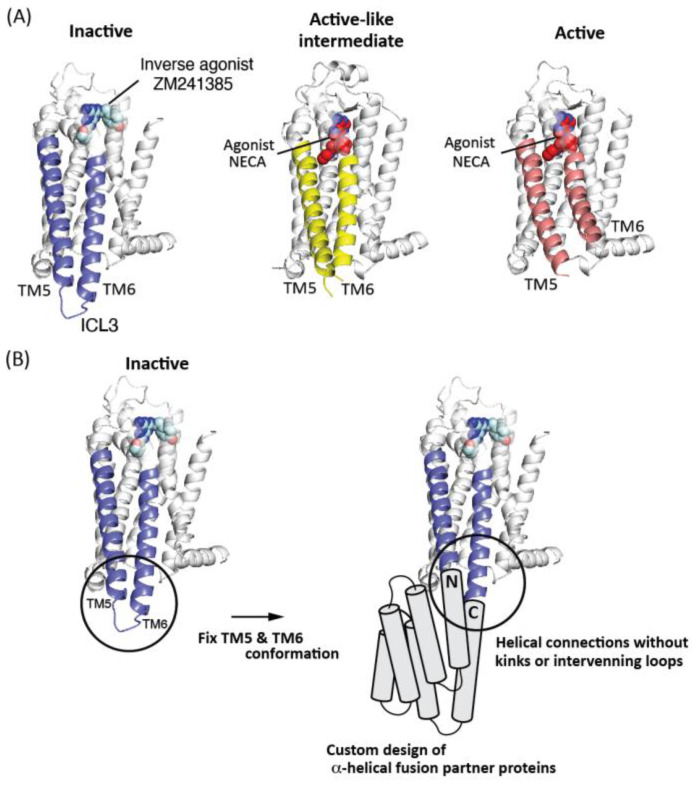
Strategy for state-targeting stabilization of GPCR, using de novo designed fusion partner proteins. (**A**) Multiple conformational states of A_2A_R. From left, the presented structures, respectively, correspond to the ones in an inactive, active-like intermediate, and activate states (PDB ID: 3PWH, 2YDV, and 5G53, respectively) [22,24,25]. The structure in the inactive state binds to the inverse agonist ZM241385, and the structures in the active-like intermediate, and active states bind to the agonist NECA. ZM241385 and NECA are shown in a sphere model. TM5 and TM6 are colored in blue for the inactive state, yellow for the active-like intermediate state, and pink for the active state. The loop connecting TM5 and TM6 is called ICL3. TM6 with TM5 exhibits large conformational changes upon the state transitions. (**B**) Our strategy for the state-targeting stabilization of A_2A_R. We fix the TM5 and TM6 conformation in a targeted state through the fusion strategy. To this end, we design α-helical proteins, which can be fused into A_2A_R in the targeted state through straight helical connections without kinks or intervening loops. In this work, we tested this idea by stabilizing the inactive state.

In this study, we designed fusion partner proteins customized for stabilizing one of the class A GPCRs, adenosine A_2A_ receptor (A_2A_R), in an inactive state [21,22]. A_2A_R plays important physiological roles, such as the modulation of motor, vascular control, and immunosuppression; therefore, A_2A_R is a drug target for various diseases, including Parkinson’s disease, heart failure, and cancer [23]. Class A GPCRs are the largest subfamily of GPCRs, and the receptors in the class have been suggested to undergo large conformational changes in TM6 associated with TM5 upon the state transitions (Figure 1A). Therefore, we stabilized A_2A_R in the inactive state by making a fusion with de novo designed proteins customized to fix the conformation of the two helices in the inactive state (Figure 1B).

## 2. Results

### 2.1. Computational Design of α-Helical Fusion Partner Proteins

**Figure 2:**
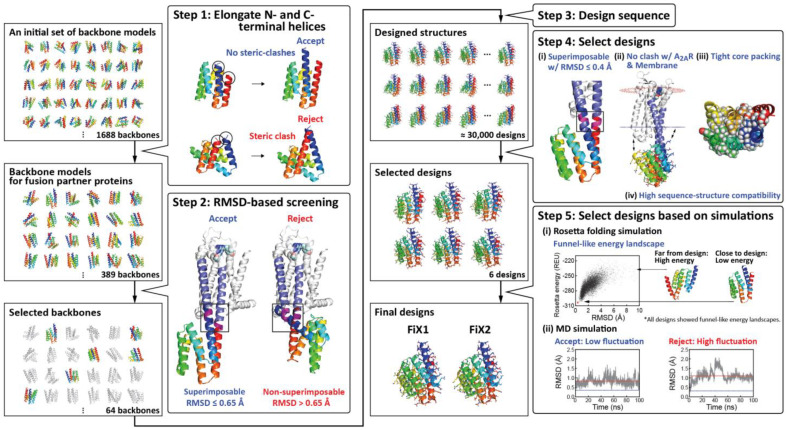
Computational protocol for designing fusion partner proteins. As an initial set of backbone structures, 1688 globular all-α backbone structures with six helices, extracted from the previously created all-α backbone structure library [20], were used. (**Step 1**) For the initial set, the N- and C- terminal helices of the structures were elongated to fuse into A_2A_R through TM5 and TM6. Then, 389 backbone structures whose helices were elongated without steric clash were selected. (**Step 2**) Backbone structures whose elongated N- and C- terminal helices were well-superimposable (the main-chain root mean square deviation (RMSD) for the fused region, equal to or less than 0.65 Å) to TM5 and TM6 of the inactive state A_2A_R structure were selected. (**Step 3**) For each selected backbone structure, amino acid sequences that stabilize the backbone structure were designed. (**Step 4**) Six designed structures were selected on the basis of the following criteria: the designs are superimposable to A_2A_R with main-chain RMSD values less than and equal to 0.4 Å for the fused region, have tight core packing [27] and high sequence-structure compatibility [17] and can be fused into A_2A_R without steric crash with A_2A_R and the membrane. (**Step 5**) Two designed proteins that exhibited funnel-shaped energy landscapes in Rosetta folding simulations [17] and whose N- and C- terminal helices show low fluctuation in molecular dynamics (MD) simulations were selected. In the MD simulations, blue and red lines show initial and averaged main-chain RMSD values, respectively. Details are described in the Results and Materials and Methods.

We assumed that the TM5 and TM6 conformation could be fixed in a specific state through straight helical connections between a fusion partner protein and A_2A_R. Therefore, we sought to design α-helical protein structures de novo, of which the N- and C-terminal helices are, respectively, connected to TM5 and TM6 of an inactive state A_2A_R structure (PDB ID: 3PWH; this structure is bound to the inverse agonist ZM241385 [22]) without any kinks or intervening loops (Figure 2) (details are described in the Materials and Methods).

Using 1688 globular all-α backbone structures with six helices [20] whose N- and C-terminal helices are close to each other, we elongated the N- and C-terminal helices by seven residues, respectively, to fuse with TM5 and TM6 of the A_2A_R inactive structure. We then selected a set of 389 backbone structures whose terminal helices were elongated without steric clash. From the generated set, we selected backbone structures whose N- and C- terminal helices are well-superimposable to TM5 and TM6 in the inactive-state A_2A_R structure by calculating root mean square deviation (RMSD) values for the main chain of superimposed residues.

Next, we designed amino-acid sequences that stabilize each of the selected backbone structures by carrying out the cycles of amino acid sequence optimization and optimization of the entire structure [26]. Among the resulting designs with tight core packing [27] and high compatibility between the local sequence and structure [17], the designs whose N- and C-terminal helices were better superimposable to TM5 and TM6 were selected. Note that the designed structures that were inside of the predicted membrane region or had clashes with A_2A_R after the fusion with A_2A_R were discarded. (The positional information of the membrane was obtained from the Orientation of Proteins in Membrane (OPM) database (https://opm.phar.umich.edu, accessed on 23 September 2021 [28]).

Next, we selected the designed proteins that exhibited funnel-shaped energy landscapes in Rosetta ab initio folding simulations [17]. Among the selected designs, we further selected those whose N- and C- terminal helices exhibited low fluctuation in molecular dynamics (MD) simulations for experimental characterization. Finally, the designed proteins, FiX1 and FiX2 (FiX stands for a **F**usion partner protein customized for **i**nactivation and e**X**tra stabilization), were selected (one of the residues in FiX2 was mutated manually using Foldit [29]. See the Material and Methods).

### 2.2. Experimental Characterization of FiX1 and FiX2

**Figure 3:**
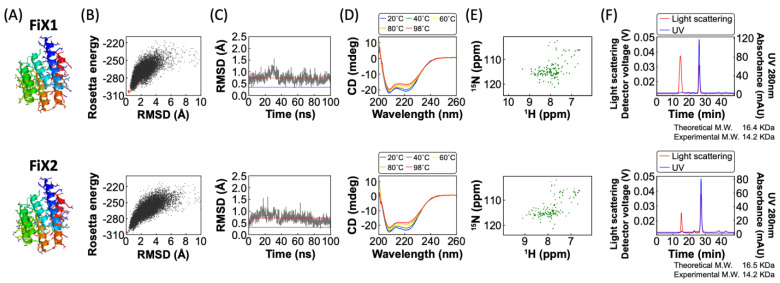
Characterization of the de novo designed fusion partner proteins without A_2A_R. De novo designed fusion partner proteins, FiX1 (upper) and FiX2 (lower). (**A**) Computational models. (**B**) The energy landscape of each designed protein obtained from Rosetta folding simulations [17]. Each dot represents the lowest energy structure obtained from an independent trajectory starting from an extended chain (black) or the design model (red), the x-axis shows the Cα RMSD from the design model, and the y-axis shows the Rosetta full-atom energy. (**C**) Structural fluctuations of the N- and C-terminal helices in the MD simulation, starting from each design model. The main-chain RMSD of the N- and C- terminal helices of each snapshot structure during a MD trajectory against TM5 and TM6 of A_2A_R is shown along the time course. (Red and blue lines are the averaged and initial RMSD values, respectively.) (**D**) Far-ultraviolet CD spectra at various temperatures from 20 to 98 °C. (**E**) Two-dimensional ^1^H–^15^N HSQC spectra at 25 °C and 600 MHz (in parts per million, p.p.m). (**F**) Size-exclusion chromatograms combined with multi-angle light scattering (SEC-MALS) demonstrate that these designed proteins are monomeric in solution. M.W. stands for molecular weight.

We experimentally characterized the de novo designed proteins, FiX1 and FiX2, without A_2A_R Table S2. These two designs were expressed in *Escherichia coli* and purified using a Ni-NTA column. Both of the designs were found to be well expressed and highly soluble, and are then characterized via circular dichroism (CD) spectroscopy, size-exclusion chromatography combined with multi-angle light scattering (SEC-MALS), and ^1^H–^15^N heteronuclear single quantum coherence (HSQC) nuclear magnetic resonance (NMR) spectroscopy (Figure 3). Both designs showed CD spectra of all-α proteins from 25 to 98 °C, were monomeric in SEC-MALS, and showed well-dispersed sharp NMR peaks. These results indicate that the designs fold into unique α-helical structures as monomers with high thermal stability.

### 2.3. Experimental Characterization of A_2A_R Fused with FiX1 and FiX2

The genes encoding the wild-type A_2A_R (A_2A_R WT) and chimeras with fusion partner proteins, A_2A_R fused with FiX1 (A_2A_R–FiX1), FiX2 (A_2A_R–FiX2), and BRIL (A_2A_R–BRIL), were constructed and expressed in yeast as described previously (for each construct, a red fluorescent protein (RFP) was appended at the C-terminus) Table S3 [30,31]. It is known that the innate instability of GPCRs gives rise to low yields upon detergent extraction from the membrane. As one of the stability metrics, we studied the solubilization efficiency upon detergent extraction (the ratio of the fluorescent intensity after solubilization using n-decyl-D-maltopyranoside (DM) over that before solubilization).

**Figure 4:**
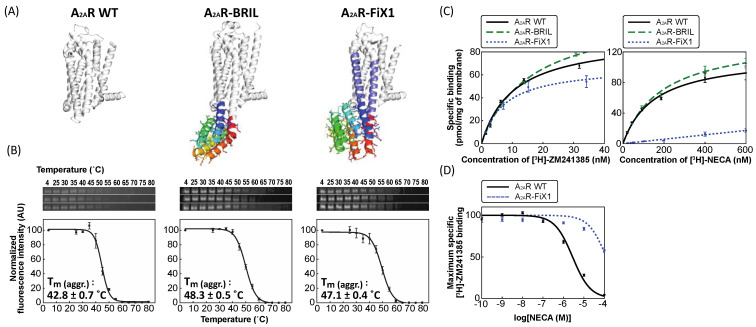
Experimental characteristics of A_2A_R fused with or without fusion partner proteins. (**A**) Crystal structures of A_2A_R WT (PDB ID: 3VG9) [22], A_2A_R–BRIL (PDB ID: 4EIY) [12], and the computational model of A_2A_R–FiX1. (**B**) (**Top**) Monomer bands in the clear-native PAGE for each A_2A_R sample heated at various temperatures. (**Bottom**) The fluorescence intensities of the gel images for each A_2A_R sample with temperature. The thermal transition from soluble to aggregated states was fitted (solid line) to obtain the midpoint temperature, Tm (aggr.) (**C**) Saturation binding curves of [^3^H]-ZM241385 (**left**) and [^3^H]-NECA (**right**) to A_2A_R WT (solid line), A_2A_R–BRIL (dashed line), and A_2A_R–FiX1 (dotted line). (**D**) Inhibition of [^3^H]-ZM241385 binding to A_2A_R WT (solid line) and A_2A_R–FiX1 (dotted line) by NECA. The binding of [^3^H]-ZM241385 in the absence of NECA was set to 100%. All measurements were carried out three times independently; dots show the average and whiskers show the s.e.m. for *n* = 3.

A_2A_R–FiX1 showed significantly improved solubilization efficiency compared to the wild-type; the efficiency was greater than that of A_2A_R–FiX2 (Table 1). Therefore, we further studied the stability of A_2A_R–FiX1 by measuring the apparent melting temperatures in the clear-native polyacrylamide gel electrophoresis (CN–PAGE) method [31]. The melting temperature was found to be significantly increased (Figure 4B), which is consistent with the solubilization efficiency results. For comparison, the solubilization efficiency and melting temperature for A_2A_R–BRIL were also measured; these values were comparable to those of A_2A_R–FiX1 (see Discussion).

Next, we investigated the ligand-binding affinities of A_2A_R WT, A_2A_R–BRIL, and A_2A_R–FiX1 using the radioligands of an inverse agonist [^3^H]-ZM241385 and an agonist [^3^H]-NECA (see Methods). The binding affinities of A_2A_R WT and A_2A_R–BRIL against ZM241385 were similar to each other, and those against NECA were also similar to each other (the obtained equilibrium dissociation constants (*K_d_*) of A_2A_R WT and A_2A_R–BRIL against ZM241385 were 10.5 nM and 15.7 nM, respectively; *K_d_* values of A_2A_R WT and A_2A_R–BRIL against NECA were 161.5 nM and 193.1 nM, respectively) (Table 2 and Figure 4C). However, the binding affinity of A_2A_R–FiX1 against ZM241385 was similar to those of A_2A_R WT and A_2A_R–BRIL, but significant binding against NECA was not observed (Table 2 and Figure 4C). Moreover, we investigated the binding affinity of A_2A_R–FiX1 to NECA using the inhibition assay, which was approximately 100-times lower than that of A_2A_R WT (Figure 4D).

For A_2A_R, the correspondence between the ligand-binding states and the conformational states has been well studied: the binding of the inverse agonist ZM241385 shifts the conformational equilibrium to the inactive state, and that of the agonist NECA shifts the equilibrium to the active state [32,33]. Therefore, these results indicate that the shifting of the conformational equilibrium of A_2A_R toward the inactive state by fusion with FiX1 was successful. The experimental structure information of A_2A_R–FiX1 is required to further support our conclusion.

## 3. Discussion

We succeeded in rationally designing a fusion partner protein, which thermally stabilized one of the class A GPCRs, A_2A_R, and stabilized it in an inactive state. We carried out the custom-made de novo design of fusion partner proteins, of which the N- and C- terminal helices are, respectably, connectable to the TM5 and TM6 in the inactive state without kinks or intervening loops. The de novo designed fusion partner proteins FiX1 and FiX2 were found to fold as monomers with high thermal stability. The fusion of A_2A_R with FiX1 was found to be not only thermally stabilized but also stabilized in the inactive state, as we designed.

We expected that A_2A_R–FiX1 would be more stable than A_2A_R–BRIL, since the melting temperature of FiX1 is over 98 °C in circular dichroism, which is far more than that of BRIL, around 65 °C [34]. However, the apparent melting temperatures of A_2A_R–FiX1 and A_2A_R–BRIL were almost the same. This suggests that the overall stability of the A_2A_R chimera is determined by the transmembrane helices or the other loops rather than the ICL3 region; therefore, the stabilization by making fusion proteins may be saturated.

Previous fusion partner strategies have used naturally occurring proteins or their mutants as fusion partner proteins. However, the number of naturally occurring proteins is limited, and their structures have been optimized during evolution to express their functions. Among naturally occurring protein structures, the ones in which the terminal helices are in close distances would be readily found. However, for fusion partner proteins to be connected to TM5 and TM6 in a specific state using straight helices, the terminal helices must have a specific distance, angle, and helical cycle. It would be difficult to find naturally occurring proteins whose terminal helix geometries exactly match all the three conditions. Moreover, most naturally occurring proteins are not stable; therefore, their folding ability can be impaired by only a few mutations. In contrast, our de novo protein design approach allows us to create stable proteins with specific helix geometries and high stability, without experimental trial and error.

The next question is whether our developed method can stabilize A_2A_R in other states (i.e., active-like intermediate or active states). GPCRs in the basal condition favor the inactive state over the other states [3]. Therefore, stabilization of A_2A_R in the other states may be more difficult than stabilization in the inactive state. Nevertheless, our success in the stabilization in the inactive state indicates that our developed method has the potential for stabilization of A_2A_R in the other states and further for the state-targeting stabilization of other GPCRs.

## 4. Materials and Methods

### 4.1. Selection of Backbone Structure Models for Fusion Partners

1688 backbone structures were extracted from the previously created all-α backbone structure library with six helices [20] with the following restrictions: (1) the maximum consecutive buried residues (a residue with accessible surface area < 5.0 Å^2^ calculated using FreeSASA (https://freesasa.github.io, accessed on 23 September 2021) [35] with a probe of radius 3.0 Å is regarded as buried) in a structure is less than 4, (2) the distance between the N- and C-terminal Cα atoms is less than 12.0 Å corresponding to a rough distance between TM5 and TM6, and (3) lower radius of gyration.

Next, the N- and C-terminal helices of the extracted backbones were extended by appending seven helical residues using the RosettaRemodel protocol [36] in Rosetta software (https://www.rosettacommons.org/software, accessed on 23 September 2021). The calculations were attempted 100 times, and if 100 backbone structures were successfully generated, their averaged structure was used as a backbone structure in the following calculation; ultimately, 389 averaged backbone structures were obtained. Next, among these structures, we selected those whose terminal helices were fusible to TM5 and TM6 of the inactive state A_2A_R structure (PDB: 3PWH, obtained from the PDB OPM database [28]).

Note that the structure of 3PWH is that of a thermally stabilized mutant; however, we used the wild-type sequence with the mutation N154Q for preventing glycosylation in the experiments. To this end, main-chain RMSD values were calculated by superimposing all pairs of three consecutive residues in the N- and C- terminal helices of the backbone structure (the residues are selected from those of the residue number from 2 to 11, and those from 113 to 123, respectively) against all pairs of the three consecutive residues in TM5 and TM6 at the cytoplasmic side (the residues were selected from those of the residue number from 204 to 211, and those from 219 to 229, respectively) (See Table S4). We selected the backbone structures that were superimposable with a main-chain RMSD value less than or equal to 0.65 Å as fusible ones; 64 backbone structures were obtained.

### 4.2. Sequence Design for Further Backbone Selection

We further screened 64 backbone structures via the sequence design of each backbone structure, followed by entire structure optimization, using the FlxbbDesign protocol in Rosetta (for the score function, talaris2014 [37] was used). In the sequence design, amino acid residue types used for each residue position were restricted based on the buriedness: hydrophobic residues were used in the protein core, hydrophilic residues on the surface, and both hydrophobic and hydrophilic residues at the boundary. Cysteine was not used to prevent unintentional disulfide bond formation; histidine was not used because of its several protonation states; glycine was used for the first and last helix residues. After the sequence design, the designs whose backbone ABEGO torsion patterns (“A” indicates the alpha region of the Ramachandran plot; “B” the beta region; “G” and “E”, the positive phi region; and “O”, the cis peptide conformation [38]) were different from those of original backbone structures were discarded. We performed the design calculations 50 times independently, and then selected backbone structures from which almost all designs were successfully generated without a change in the backbone ABEGO torsion pattern. Finally, we obtained three backbone structures.

### 4.3. Sequence Design

For each of the selected three backbone structures, sequence designs were performed 10,000 times, using the design protocol described in above section, with additional restrictions for used amino acid residue types. (1) When the backbone dihedral angle was classified as G based on the ABEGO classification [38], the amino acid type of the residue was fixed to glycine; (2) serine and threonine on α-helices were not used, except for the first and last helix residues, because these residues have a tendency to bend α-helices [39]; (3) positively charged residues, lysine and arginine, were not used in the first three helix residues, based on a previous report [40].

### 4.4. Selection Criteria after Sequence Design

After the sequence design, the designs were selected by the following criteria. (1) the main-chain RMSD value between the N- and C-terminal helices and TM5 and TM6 of A_2A_R is less than or equal to 0.4 Å and (2) designs with tight-core packing calculated by Rosetta Holes [27] (more than 0 and less than 2.0) and Packstat (more than 0.6) in Rosetta software (https://www.rosettacommons.org/software, accessed on 23 September 2021). Then, designed structures that had clashes with A_2A_R in a fused structure were discarded (a clash was identified by the distances between the Cα atoms of a design and A_2A_R being less than 5.5 Å). In addition, designed structures that were to be inside of the membrane were also discarded (it is not allowed that even one of the atoms of a designed protein in a fused structure is in the membrane region; the membrane region was obtained from the Orientation of Proteins in Membrane (OPM) database [28]). Moreover, designs with high compatibility between the local sequence and structure were selected in the following manner. For each nine-residue frame of a designed protein, 200 nine-residue fragments were collected from a non-redundant set of X-ray structures based on the sequence similarity and secondary structure prediction. Then, for each frame, the RMSD of the local structure against each of the 200 fragments was calculated. Designs were ranked according to the summation of the log-ratio of the fragments, for which the RMSD was less than 1.5 Å, across all nine-residue frames, and six design sequence with high values were selected.

### 4.5. Rosetta Folding Simulation

Energy landscapes of the designed sequences were obtained from Rosetta folding simulations [17]. For each amino acid sequence of designed proteins, 10,000 predicted structure models were generated starting from a completely extended structure. Furthermore, 200 energy-minimized structure models were generated starting from each of the designed protein structures. The energy landscape of each designed structure was evaluated by the shape of the scatter plot of the Rosetta score of the generated models versus the corresponding RMSD values to the designed structure. We confirmed that the predicted energy landscapes for all the designs were funnel-like.

### 4.6. Molecular Dynamics (MD) Simulation

MD simulations were performed to select designed structures whose N- and C-terminal helices did not fluctuate significantly in the simulations. Main-chain RMSD values were calculated by superimposing three consecutive residues in the N- and C- terminal helices of each snapshot structure generated during an MD trajectory against three consecutive residues in TM5 and TM6. The positions for the three consecutive residues were those used in the RMSD-based screening calculation (Table S4). The designs with average RMSD values of more than 0.75 Å or unexpected hydrophilic interactions in the MD simulation trajectories were discarded.

The AMBER16 software suite [41] was used to perform the MD simulations. Hydrogen atoms were added using the LEaP module in AMBER16, after removing those from the design models. A box with a 12 Å buffer of water models around the protein model in each direction was created. TIP3P [42] and AMBER ff99SB force fields [43] were used as the water model and protein force field, respectively. Periodic boundary conditions were set at a cut-off distance of 10 Å. Long-range electrostatic interactions were treated using the particle mesh Ewald method.

At the beginning of the simulations, energy minimization of the solvent was performed with harmonic restriction for protein atoms, and subsequently energy minimization without restriction was performed. Next, the temperature of the system was gradually increased from 0 to 300 K in 100 ps in an NVT ensemble with a Langevin thermostat and harmonic positional restriction for the protein atoms. After the heating step, a 100 ns MD simulation was performed at 1 atm at 300 K in an NPT ensemble with isotropic position scaling, setting one step as 0.002 ps.

### 4.7. A Manual Mutation Using Foldit

The Tyr residue at the position 67 in FiX2 was manually mutated to Leu using Foldit [29] to optimize the core packing.

### 4.8. Experiments of De Novo Designed Fusion Partner Proteins: Protein Expression and Purification

Plasmids with FiX1 or FiX2 DNA sequences between the NdeI and XhoI restriction sites in pET21b vectors were purchased from FASMAC (Kanagawa, Japan). *E. coli* BL21 Star (DE3) competent cells were transformed with the plasmids and cultured in MJ9 minimal media containing ^15^N-labeled ammonium sulfate as a nitrogen source, and ^15^N-labeled FiX1 and FiX2 were expressed. After the cells were spun down, they were suspended in phosphate-buffered saline (PBS) buffer, 137 mM NaCl, 2.7 mM KCl, 10 mM Na_2_HPO_4_, and 1.8 mM KH_2_PO_4_ at pH 7.4 with BugBuster (EMD Millipore Corp., Billerica, MA, USA), protease inhibitor, lysozyme, and deoxyribonuclease.

From the cell lysates, FiX1 and FiX2 samples with a His-tag at C-terminus were purified using a Ni-NTA column. The purified samples were dialyzed against PBS buffer at pH 7.4. The purity of the FiX1 and FiX2 samples was confirmed via SDS-PAGE (Figure S1) and mass spectrometry.

### 4.9. Experiments of De Novo Designed Fusion Partner Proteins: Circular Dichroism (CD)

CD spectra were measured using J-1500 KS (JASCO Corp., Tokyo, Japan). By heating the samples from 20 to 98 °C at a rate of 1 °C per min, far-UV CD spectra were measured from 260 to 200 nm at various temperatures of 20, 40, 60, 80, and 98 °C using 10 μM FiX1 and FiX2 samples in PBS buffer (pH 7.4) in a 1-mm path length cuvette.

### 4.10. Experiments of De Novo Designed Fusion Partner Proteins: Size Exclusion Chromatography Combined with Multi-Angle Light Scattering (SEC-MALS)

SEC-MALS measurements were performed using a miniDAWN TREOS static light scattering detector (Wyatt Technology Corp., Santa Barbara, California, USA) and a high-performance liquid chromatography (HPLC) system (1260 Infinity LC, Agilent Technologies, Santa Clara, CA, USA). Approximately 180 μM FiX1 and FiX2 samples in PBS buffer (pH 7.4) were injected into a Superdex 75 increase 10/300 GL column (GE Healthcare) equilibrated with PBS at a flow rate of 0.5 mL/min. Sample concentrations were evaluated based on the absorbance at 280 nm detected by using HPLC system. Static light scattering data at the angles of 43.6°, 90.0°, and 136.4° were obtained using a 659 nm laser. The data were analyzed using ASTRA software (https://store.wyatt.com/shop/viscostar/viscostar-iii/astra-software/, accessed on 23 September 2021) (version 6.1.2, Wyatt Technology Corp., Santa Barbara, California, USA) with a dn/dc value of 0.185 mL/g.

### 4.11. Experiments of De Novo Designed Fusion Partner Proteins: 2D ^1^H–^15^N HSQC Measurement

For ^15^N-labeled FiX1 and FiX2 samples of 400 to 600 μΜ in 90% H_2_O/10% D_2_O PBS buffer (pH 7.4), 2D HSQC NMR spectrum measurements were performed using a JNM-ECA 600 MHz spectrometer (JEOL, Tokyo, Japan). The obtained NMR spectra were analyzed using the Delta NMR software (https://nmrsupport.jeol.com/Software, accessed on 23 September 2021) (version 5.2.1, JEOL, Tokyo, Japan).

### 4.12. Experiments of A_2A_R-Designed Fusion Partner Proteins: DNA Construction

The coding sequence of the human adenosine A_2A_ receptor (A_2A_R) from residues 1−316 was amplified by using the polymerase chain reaction (PCR) method, in which N154 was replaced by Q to eliminate N-linked glycosylation [44]. The DNA fragment was inserted into the plasmid pDDGFP-2 [45], including TagRFP-His8 at the C-terminus [31]. The intracellular loop 3 (ICL3) of the A_2A_R (denote A_2A_R WT) was replaced with FiX1 or FiX2. The residue numbers of the de novo designed fusion partner proteins refer to the original amino acid sequences on the pET21b vectors (Table S1).

### 4.13. Experiments of A_2A_R-Designed Fusion Partner Proteins: Solubilization Efficiency

Wild-type A_2A_R and its variants (A_2A_R–BRIL, A_2A_R–FiX1, and A_2A_R–FiX2) were expressed in *Saccharomyces cerevisiae* strain FGY217, and the membranes were prepared as described previously [31]. Briefly, membranes were resuspended in a solubilization buffer (50 mM Tris, 120 mM NaCl, 20% glycerol, and 1 μg/mL 6-amidinonaphthalen-2-yl 4-guanidinobenzoate bis (methanesulfonate) (AFP) (Alfresa Pharma Corp., Osaka, Japan); pH 8.0). The membrane suspension (5 mg/mL) was solubilized using n-decyl-β-D-maltopyranoside (DM) (final concentration, 1%) (Anatrace, Maumee, OH, USA) for 30 min at 4 °C. The red fluorescent protein (RFP) intensity was measured before and after solubilization at 595 nm (excitation at 535 nm) using a FilterMax F5 microplate reader (Molecular Devices, Sunnyvale, CA, USA). The solubilization ratio was evaluated as the RFP intensity of the unpurified A_2A_R–RFP divided by that of the whole membrane protein mixture soon after DM solubilization.

### 4.14. Experiments of A_2A_R-Designed Fusion Partner Proteins: Clear-Native PAGE

We evaluated the apparent melting temperatures of the wild-type A_2A_R and its variants (A_2A_R–BRIL, A_2A_R–FiX1) solubilized in 1% n-dodecyl β-D-maltopyranoside (DDM; Anatrace, Maumee, OH, USA) containing 0.2% cholesterol hemisuccinate (CHS; Sigma-Aldrich, Saint Louis, MO, USA), using clear native polyacrylamide gel electrophoresis (CN-PAGE) with modified Coomassie Brilliant Blue G-250 (mCBB) stain [31]. The samples were fused with RFP at the C-terminus, exhibiting fluorescence at 595 nm.

The samples were heated at each prescribed temperature for 5 min (the temperatures were prescribed in the range of 25–80 °C), and then immediately cooled on ice. CN-PAGE was performed using 10% Tris-glycine separation gel applied to the treated samples with CN-PAGE buffer (200 mM Tris-HCl, 20% glycerol, 1.0% mCBB, and 1.0% DDM; pH 8.6) at a ratio of 1:1. The samples on the gel were visualized (i.e., gel imaging was performed) using FUSION SOLO 7S (Vilber–Lourmat, Marne-la-Vallée, France) after a 5-s exposure to green light at 530 nm with a 655 nm cutoff filter.

The melting temperatures of the samples were determined from the fluorescence intensities of the monomeric bands on the CN-PAGE gel. The normalized fluorescence intensity was calculated by dividing the fluorescence intensity of the monomeric bands after heating by that before heating and is represented as a percentage. The obtained intensities of the monomeric bands were quantified using the ImageJ software (https://imagej.nih.gov/ij, accessed on 23 September 2021). The melting temperatures were calculated using GraphPad Prism 4.0 (GraphPad Software, San Diego, CA, USA) as previously described [31].

### 4.15. Experiments of A_2A_R-Designed Fusion Partner Proteins: Radioligand Binding Assay

Radioligand binding assays were performed using yeast cell membranes expressing the wild-type A_2A_R and its variants (A_2A_R–BRIL and A_2A_R–FiX1). The protein concentrations of the membranes were determined by using the bicinchoninic acid method (Thermo Fisher Scientific, Waltham, MA, USA) with bovine serum albumin as a standard. All experiments were performed in triplicate (independent expressions). For the saturation-binding assay, 10 µg of membranes were incubated (3 h on ice) with the inverse agonist [^3^H]-ZM241385 (American Radiolabeled Chemicals, Saint Louis, MO, USA) at concentrations ranging from 5 to 80 nM and the agonist [^3^H]-NECA (PerkinElmer, Waltham, MA, USA) at concentrations ranging from 25 to 600 nM. Non-specific binding was determined in the presence of 10 μM ZM241385 (Sigma-Aldrich, St. Louis, MO, USA) and 100 μM NECA (Sigma-Aldrich, St. Louis, MO, USA), respectively.

For the competition-binding assay, 10 µg of membranes were incubated with 20 nM of [^3^H]-ZM241385 and unlabeled NECA at concentrations ranging from 1 nM to 100 µM of [^3^H]-NECA for 3 h on ice. The unbound ligand was removed by rapid vacuum filtration over GF/F filters (GE Healthcare, Chicago, IL, USA). Filtration was performed using a MINI-VAC (Yamato Scientific, Tokyo, Japan) at room temperature. The filters were washed twice with solubilization buffer. After adding 5 mL of Filter-Count (PerkinElmer, Waltham, MA, USA), radioactivity was measured using an LSC-6100 liquid scintillation counter (Hitachi ALOKA Medical, Tokyo, Japan). The collected data were analyzed by using a nonlinear regression-fitting program in GraphPad Prism 8 (GraphPad Software, San Diego, CA, USA).

## Figures and Tables

**Table 1:** Solubilization efficiencies of A_2A_R fused with or without fusion partner proteins. Results are reported as mean ± standard deviation for *n* = 3 independent measurements.

Construct	Solubilization Efficiency (%)
A_2A_R WT	24 ± 10
A_2A_R–BRIL	57 ± 19
A_2A_R–FiX1	59 ± 21
A_2A_R–FiX2	42 ± 17

**Table 2:** Dissociation constants (*K_d_*) of A_2A_R fused with or without fusion partner proteins by saturation binding assay. N.D. stands for not detected. Results are reported as the mean ± standard deviation for an *n* = 3 independent assay.

Construct	*K*_d_ (nM)	
	ZM241385	NECA
A_2A_R WT	10.5 ± 0.3	162 ± 44
A_2A_R–BRIL	15.7 ± 0.6	191 ± 22
A_2A_R–FiX1	7.3 ± 0.5	N. D.

## Data Availability

The data presented in this study are available on request from the corresponding author.

## Supplementary Materials

The following are available online at https://www.mdpi.com/article/10.3390/ijms222312906/s1. Figure S1, SDS-PAGE results for FiX1 and FiX2. Table S1, Sequence regions for A2AR and fusion partner proteins in A2AR chimeras. Table S2, Amino acid sequences of FiX1 and FiX2. Table S3, Amino acid sequences of A2AR fused with FiX1 and FiX2. Table S4, Residues used for superposition between A2AR and de novo designed fusion partner proteins.
