# Supplementary material for: State-Targeting Stabilization of Adenosine A2A Receptor by Fusing a Custom-Made De Novo Designed α-Helical Protein
Source: Int J Mol Sci. 2021 Nov 29;22(23):12906. doi: 10.3390/ijms222312906 (PMC8657880; doi:10.3390/ijms222312906)
Supplement: Supplementary file 1 [file ijms-22-12906-s001.zip › ijms-1414223-supplementary.pdf]

Supplementary Information for

State-Targeting Stabilization of Adenosine A_{2A} Receptor by Fusing a Custom-Made De Novo Designed α-Helical Protein

Masaya Mitsumoto[1,2], Kanna Sugaya[3], Kazuki Kazama[3], Ryosuke Nakano[3], Takahiro Kosugi[1,2,4], Takeshi Murata[3,5,*] and Nobuyasu Koga[1,2,4,*]

[1] Department of Structural Molecular Science, School of Physical Sciences, SOKENDAI, The Graduate University for Advanced Studies, Shonan Village, Hayama, Kanagawa 240-0193, Japan.
[2] Research Center of Integrative Molecular Systems, Institute for Molecular Science (IMS), National Institutes of Natural Sciences (NINS), Okazaki, Aichi 444-8585, Japan.
[3] Department of Chemistry, Graduate School of Science, Chiba University, Chiba, Chiba 263-8522, Japan.
[4] Protein Design Group, Exploratory Research Center on Life and Living Systems (ExCELLS), National Institutes of Natural Sciences (NINS), Okazaki, Aichi 444-8585, Japan.
[5] Membrane Protein Research Center, Chiba University, Chiba 263-8522, Japan

[*]e-mail: t.murata@faculty.chiba-u.jp or nkoga@ims.ac.jp

Supplementary Figures

1. **SDS-PAGE results for FiX1 and FiX2**

Supplementary Tables

1. **Sequence regions for A_{2A}R and fusion partner proteins in A_{2A}R chimeras**
2. **Amino acid sequences of FiX1 and FiX2**
3. **Amino acid sequences of A_{2A}R fused with FiX1 and FiX2**
4. **Residues used for superposition between A_{2A}R and *de novo* designed fusion partner proteins**

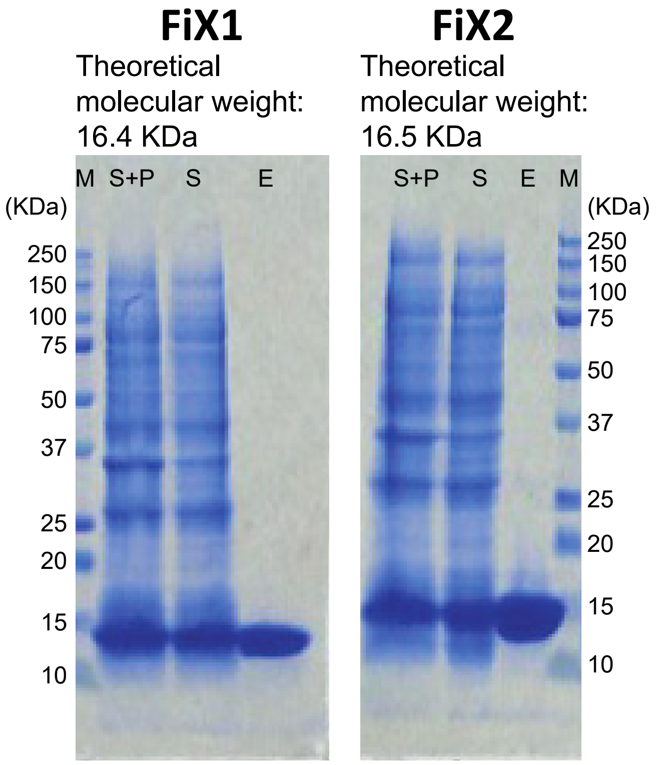

Figure S1 | SDS-PAGE results for FiX1 and FiX2

M, S+P, S and E indicate marker, a mixture of supernatant (S) and pellet (P) after cell lysates; S, supernatant after centrifugation of cell lysates; and E, elution after Ni purification, respectively.

Table S1 | Sequence regions for $A_{2A}R$ and fusion partner proteins in $A_{2A}R$ chimeras

Construct	Truncated regions in $A_{2A}R$	Inserted regions in fusion partner proteins
**$A_{2A}R$-BRIL**	K209 - G218	A1 – L106
**$A_{2A}R$-FiX1**	K209 - R220	R10 - E119
**$A_{2A}R$-FiX2**	K209 - E219	R10 - E118

Table S2 | Amino acid sequences of FiX1 and FiX2

Computationally designed amino acid sequences are described in uppercase and residues added to allow expression and purification are described in lowercase.

Construct	Sequence
**FiX1**	mGEEEEERRRRLLELLKRIAELLERGDLEEALKLVKKLAKEQGRQEIIDYIEEVLRLYQEGNREEARKLLE ELLRRLEKEGDTEFRELIRIILEFLELEERGDLEEAKKLARELKKQVDEQEKRLGlehhhhhh
**FiX2**	mGEEEEERRRRLLELLERIARLLKRGDLEEALKLVKKLAKEQGEQEIIDFIEEVLRLYQEGNREQARELLE RLLRNLEKRGNQDFRNLIEIILRILELEQRGNQEEIKKLAEELRREVEERKRKLGlehhhhhh

Table S3 | Amino acid sequences of A_{2A}R fused with FiX1 and FiX2

Amino acid sequences of fusion partner proteins, FiX1 and FiX2, are highlighted by underlines. Glycine spacer + TEV protease recognition site + TagRFP + 8xHis-tag (lowercase) is added at the C-terminal of each designed protein.

Construct	Sequence
**A_{2A}R-FiX1**	MPIMGSSVYITVELAIAVLAILGNVLVCWAVWLNSNLQNVTNYFVVSLAAADIAVGVLAIPFAITISTG<br>FCAACHGCLFIACFVLVLTQSSIFSLLAIAIDRYIAIRIPLRYNGLVTGTRAKGIIAICWVLSFAIGLT<br>PMLGWNNCGQPKEGKQHSQGCGEGQVACLFEDVVPMNYMVYFNFFACVLVPLLLMLGVYLRIFLAARRQ<br>LRLLELLKRIAELLERGDLEEALKLVKKLAKEQGRQEIIDYIEEVLRLYQEGNREEARKLLEELLRRLE<br>KEGDTEFRELIRIILEFLELEERGDLEEAKKLARELKKQVDEARSTLQKEVHAAKSLAIIVGLFALCWL<br>PLHIINCFTFFCPDCSHAPLWLMYLAIVLSHTNSVVNPFIYAYRIREFRQTFRKIIRSHVLRQQEPFKA<br>genlyfqgvskgeelikenmhmklymegtvnnhhfkctsegegkpyegtqtmrikvveggplpfafdil<br>atsfmygsrtfinhtqgipdffkqsfpegftwervttyedggvltatqdtslqdgcliynvkirgvnfp<br>sngpvmqkktlgweantemlypadgglegrsdmalklvggghlicnfkttyrskkpaknlkmpgvyyvd<br>hrlerikeadketyveqhevavarycdlpsklghklnhhhhhhhh
**A_{2A}R-FiX2**	MPIMGSSVYITVELAIAVLAILGNVLVCWAVWLNSNLQNVTNYFVVSLAAADIAVGVLAIPFAITISTG<br>FCAACHGCLFIACFVLVLTQSSIFSLLAIAIDRYIAIRIPLRYNGLVTGTRAKGIIAICWVLSFAIGLT<br>PMLGWNNCGQPKEGKQHSQGCGEGQVACLFEDVVPMNYMVYFNFFACVLVPLLLMLGVYLRIFLAARRQ<br>LRLLELLERIARLLKRGDLEEALKLVKKLAKEQGEQEIIDFIEEVLRLYQEGNREQARELLERLLRNLE<br>KRGNQDFRNLIEIILRILELEQRGNQEEIKKLAEELRREVERARSTLQKEVHAAKSLAIIVGLFALCWL<br>PLHIINCFTFFCPDCSHAPLWLMYLAIVLSHTNSVVNPFIYAYRIREFRQTFRKIIRSHVLRQQEPFKA<br>genlyfqgvskgeelikenmhmklymegtvnnhhfkctsegegkpyegtqtmrikvveggplpfafdil<br>atsfmygsrtfinhtqgipdffkqsfpegftwervttyedggvltatqdtslqdgcliynvkirgvnfp<br>sngpvmqkktlgweantemlypadgglegrsdmalklvggghlicnfkttyrskkpaknlkmpgvyyvd<br>hrlerikeadketyveqhevavarycdlpsklghklnhhhhhhhh

Table S4 | Residues used for superposition between A_{2A}R and *de novo* designed fusion partner proteins

	TM5 and TM6 in A_{2A}R	N- and C-terminal regions in FiX1 and FiX2
**A_{2A}R vs FiX1**	R206-L208 and A221-S223	R7-R9 and Q120-K122
**A_{2A}R vs FiX2**	R206-L208 and R220-R222	R7-R9 and E119-K121
